# Pixel detectors for diffraction-limited storage rings

**DOI:** 10.1107/S1600577514017135

**Published:** 2014-08-31

**Authors:** Peter Denes, Bernd Schmitt

**Affiliations:** a Advanced Light Source, Lawrence Berkeley National Laboratory, 1 Cyclotron Road, Berkeley, CA 94720, USA; b Swiss Light Source, Paul Scherrer Institut, OFLC/001, Villigen 5232, Switzerland

**Keywords:** detectors, hybrid pixel detectors, monolithic pixel detectors, diffraction-limited storage rings, RIXS, XPCS, CXDI

## Abstract

Dramatic advances in synchrotron radiation sources produce ever-brighter beams of X-rays, but those advances can only be used if there is a corresponding improvement in X-ray detectors. With the advent of storage ring sources capable of being diffraction-limited (down to a certain wavelength), advances in detector speed, dynamic range and functionality is required. While many of these improvements in detector capabilities are being pursued now, the orders-of-magnitude increases in brightness of diffraction-limited storage ring sources will require challenging non-incremental advances in detectors. This article summarizes the current state of the art, developments underway worldwide, and challenges that diffraction-limited storage ring sources present for detectors.

## Abstract 2

Two-dimensional detector improvements required to take advantage of diffraction-limited storage ring light sources are discussed.

## 1. Soft X-ray detectors   

### 1.1. Introduction   

**Figure 1:**
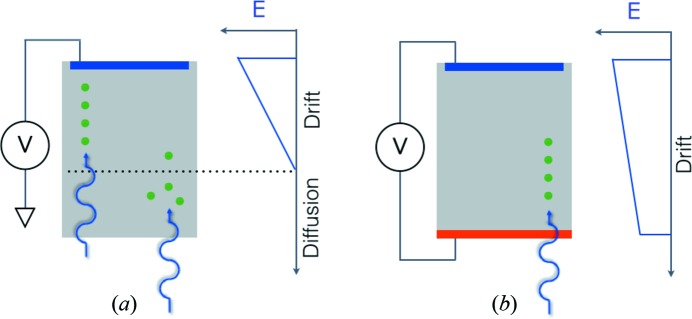
Schematic of a partially depleted (*a*) and fully depleted (*b*) semi­conductor detector.

For soft X-ray (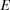 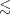 2 keV) detection, low noise is essential. Gain can be used to overcome noise, for example microchannel plates (which tend to have low quantum efficiency) or avalanche photodiodes (which are used as point detectors). In silicon, 3.6 eV are required to produce one electron/hole pair, and, with a single-photon threshold of five times the noise, noise levels of less than 100 eV are required. As a consequence, soft X-ray direct detectors tend to be monolithic (with generally smaller, hence lower capacitance, pixels than hybrid detectors, described in §2), which adds constraints on the readout electronics. To date, variants of charge-coupled devices (CCDs) have been dominant as high-efficiency two-dimensional detectors. Commercial detectors are generally thinned (tens of micrometres), back-illuminated, partially depleted CCDs. Pixel readout rates are comparatively slow (∼1 Megapixel s^−1^, due to the serial nature of CCD readout and the limited number of readout ports), although good noise can be achieved, particularly for detectors employing electron-multiplying readout. As shown in Fig. 1 ▶, for a fully depleted detector (Fig. 1*b* ▶) there are no field-free regions and all charge is collected by drift. Spatial resolution is limited by scattering, and energy resolution is limited by readout noise and conversion statistics (Fano factor). In the case of a partially depleted detector (Fig. 1*a* ▶), if the photon converts in the depleted region (as shown on the left) it behaves like a fully depleted detector. If the photon converts in the field-free region, however (as shown on the right), charge diffuses into 4π. This not only degrades spatial resolution but also effects energy resolution, as some fraction of the diffusing charge will be lost to recombination.

To overcome all of these limitations, the community has developed two CCD-based direct detectors (Denes *et al.*, 2009 ▶; Strüder *et al.*, 2010 ▶) with technical differences in the implementation, but both using thick (hundreds of micrometres) high-resistivity fully depleted silicon for high quantum efficiency and multiple readout ports for high-speed readout (∼hundreds of megapixels per second). A similar concept has also been used for hard X-ray free-electron laser (FEL) applications (Kameshima *et al.*, 2014 ▶). In the future, back-illuminated CMOS active pixel sensors, commercially produced for cellphone cameras for several years, hold the promise of performance and ease-of-use of commercial CMOS for soft X-rays (Wunderer *et al.*, 2014 ▶).

### 1.2. Brighter diffraction-limited sources   

As sources approach the soft X-ray diffraction limit, the increased brightness and coherence will necessitate advances in the technology of current detectors (*e.g.* for scanning microscopies, coherent imaging, *etc*.) to permit the higher readout rates, higher dynamic range and improved sensitivity needed to take advantage of source improvements. (For example, in ptychography the dynamic range scales as the fourth power of the probe size divided by the spatial resolution, so that higher spatial resolution requires much higher dynamic range detectors.) In addition to improvements in current detectors, (at least) two new types of soft X-ray detectors will be needed, as described below.

#### 1.2.1. Detectors for resonant inelastic X-ray scattering (RIXS)   

**Figure 2:**
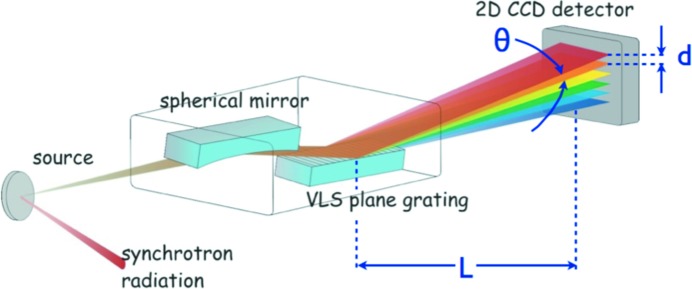
Illustration of a RIXS beamline.

RIXS is a powerful, but photon-hungry, technique gaining in popularity as sources improve. Worldwide, several new RIXS beamlines are under construction or planned. Generally targeted towards earth-abundant materials, X-ray energies of interest are typically 500–2000 eV. RIXS beamlines employ dispersive spectrometers (Fig. 2 ▶) so that energy resolution 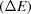 translates into X-ray angular resolution 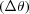. For a detector with spatial resolution *d*, and a beamline with length from spectrometer to detector of *L*, then 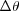 = *d*/*L*. One can then either improve detector spatial resolution or simply make the beamline longer. To date, the approach has been the latter, although there are now finally efforts on the former: seeking to improve the intrinsic pixel spatial resolution (currently tens of micrometres) by detector design, or to improve the spatial resolution by ‘software’, *i.e.* centroiding hits allowing resolution of the order of a few micrometres (Soman *et al.*, 2013 ▶). For RIXS the main detector requirements are very good position resolution in the energy-dispersive direction and large area coverage in the other direction to increase the statistics. The requirements currently can be satisfied by a size of 2–3 cm in the energy-dispersive and 7–10 cm in the other direction.

#### 1.2.2. X-ray photon correlation spectroscopy (XPCS)   

XPCS provides a means to study fluctuating systems using bright coherent X-ray sources. Interestingly, XPCS temporal resolution scales as the square of the brightness (Falus *et al.*, 2006 ▶) so that this technique benefits dramatically from increases in brightness. As with RIXS, soft X-rays are useful for studying earth-abundant materials, particularly to be able to probe in the water window (280–540 eV). Presently, fast two-dimensional detectors are used at high frame rates in order to record the time-varying speckle pattern. A diffraction-limited source will enable beams of spot size *D* in the micrometre range, and the length scale being probed ≃ *D*/*N*, where *N* is the number of speckles separation from the measured X-ray and the direct beam (and the speckle size is given by the wavelength multiplied by the distance to the detector divided by the spot size). Extending XPCS to brighter sources means that future two-dimensional detectors will need to be faster, while maintaining a large number of pixels in order to reach nanometre length scales.

With sufficient brightness, it would become possible to study chemical kinematics on a nanosecond–nanometre scale, which can reveal spatial correlations between catalytic centres. To achieve nanosecond time scales, such a detector would ‘tag’ each speckle with an X-ray pulse number (or time-stamp at as high a rate as practical), which is extremely challenging for a soft X-ray detector. As pulses on a diffraction-limited source are longer duration than on present sources, if it were possible to detect the rare events where there are two photons from the same speckle within one pulse, sub-nanosecond times could be accessed.

## 2. Detectors for the medium energy range   

Typical applications at this energy range (2–25 keV) are small-angle scattering, diffraction methods like protein crystallography (PX), powder diffraction or applications making use of the coherence of the beam such as coherent diffractive imaging (CDI) or ptychography.

Compared with today’s third-generation synchrotrons, diffraction-limited light sources offer a much smaller horizontal divergence resulting in a much higher brilliance and also a much higher coherence resulting in a higher coherent flux by up to a few orders of magnitude. Therefore, the main challenges for detectors come from applications using the coherence of the beam such as CDI or ptychography. For these applications, the incoming flux will be up to two to three orders of magnitudes higher. The biggest challenge for detectors at diffraction-limited light sources will therefore be the count rate capability. Other applications may also see an increase of flux due to sharper energy peaks in the undulator harmonics or better focusing optics both coming from a smaller horizontal divergence. The increase of flux will, however, be smaller than for applications making use of the coherence.

Other tendencies, like an increase of the beamline flux by increasing the energy bandwidth (*e.g.* by using a multilayer monochromator), will give an additional increase of a factor of ten in intensity. This is applicable for today’s synchrotrons as well as for future diffraction-limited light sources.

The detectors for the medium energy range today are usually single-photon-counting hybrid pixel detectors. The sensor material is usually silicon, providing an efficiency above 80% for energies in the range 2–14 keV assuming a 500 µm-thick sensor. Each pixel in the sensor is connected *via* bump-bonding to a channel in the readout ASIC (application specific integrated circuit) providing a charge-sensitive preamp, a shaper and a comparator incrementing a counter if the charge signal is above a user-defined threshold. The detector operates basically noise-free for thresholds set above five times the noise. Examples of this type of detector are Pilatus (Kraft *et al.*, 2009 ▶), Eiger (Dinapoli *et al.*, 2014 ▶), Medipix (Llopart *et al.*, 2010 ▶; Ballabriga *et al.*, 2011 ▶), Maxipix (Ponchut *et al.*, 2011 ▶), Excalibur (Marchal *et al.*, 2013 ▶), Lambda (Pennicard *et al.*, 2011 ▶) and IMXPAD (Berar *et al.*, 2009 ▶).

Single-photon-counting detectors today already often reach their count-rate limit, and cannot cope with a factor of ten higher flux coming from an increase in energy bandwidth of the monochromator or the up to three orders of magnitude higher coherent flux expected at diffraction-limited light sources.

Improvements in the count-rate capability by using, for example, time over threshold as a measure for the number of photons or the instant retrigger capability of Pilatus3 (Loeliger *et al.*, 2012 ▶) are possible but the count rate is still limited and necessitates very large channel-dependent count rate corrections above a few MHz. New approaches with charge-integrating front-ends are therefore necessary for diffraction-limited light sources.

### 2.1. Detectors with higher count-rate capabilities   

As noted above, the main limitation of single-photon-counting detectors is their limited count-rate capability. This limitation comes from signal pile-up where the analogue signal for two or more photons does not fall below the threshold voltage in between photons so that the photons are counted as one. Single-photon-counting detectors typically have a count-rate capability of a few MHz requiring large count-rate corrections. But also other limitations exist, like the minimum achievable pixel size due to the requirement to put a lot of electronics (preamp, shaper, comparator and counter) into a pixel and from charge sharing between pixels in the sensor; and the noise and cross-talk on the chip resulting in a minimum energy threshold of 1–1.5 keV cutting off the low energy range.

An approach which can overcome all these three limitations of single-photon-counting detectors without giving up on the single-photon sensitivity is a charge integration approach with dynamic gain switching. This approach is also the most promising choice for detectors for XFELs where a photon arrival time of 100 fs makes single-photon counting impossible. A dynamic gain switching approach has been implemented in several detectors [Gotthard (Mozzanica *et al.*, 2012 ▶), AGIPD (Becker *et al.*, 2013 ▶), Jungfrau (Mozzanica *et al.*, 2014 ▶), DSSC (Porro *et al.*, 2012 ▶), ePix (Dragone *et al.*, 2014 ▶)]. Gotthard, AGIPD, Jungfrau and ePix implement the dynamic gain switching in the readout ASIC, DSSC implements a non-linear response in the DEPFET sensor itself. The dynamic gain switching uses a high gain at low intensities to achieve single-photon sensitivity and then dynamically switches to lower gains to avoid saturation. In this way each pixel adjusts itself dynamically to the incoming number of photons.

Other approaches are realised in the mixed-mode PAD from Cornell (Koerner *et al.*, 2011 ▶) which combines a charge-integrating front-end which is discharged to prevent saturation or CSPad (Hugh *et al.*, 2011 ▶) with implemented statically selectable gains to either have single-photon resolution or a larger but still limited dynamic range.

### 2.2. The Jungfrau detector   

**Figure 3:**
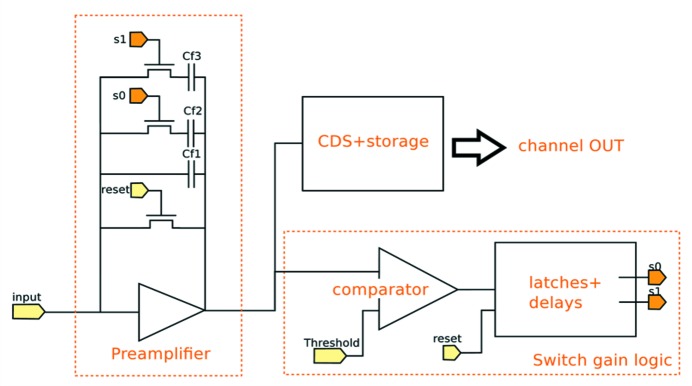
Dynamic gain-switching front-end. After reset a comparator monitors the output of the charge-integration stage and just before saturation switches in larger feedback capacitors to reduce the gain. In this way each pixel adjusts itself to the incoming number of photons.

**Figure 4:**
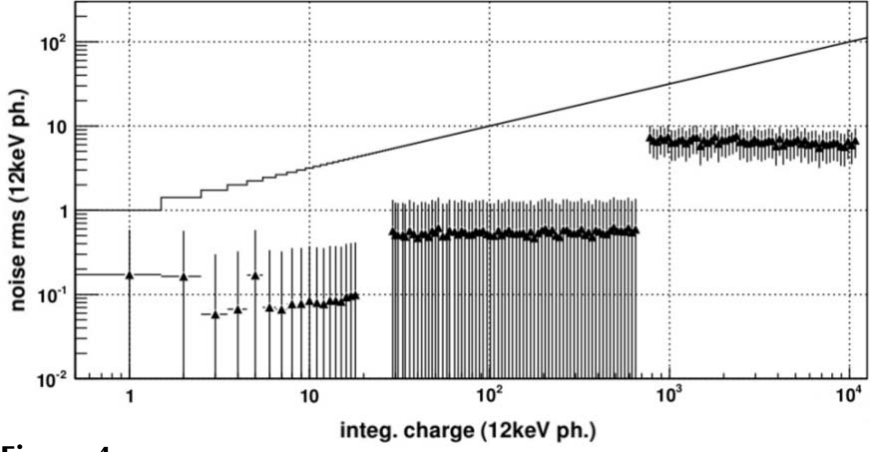
Noise (normalized to 12 keV photons) measured in Jungfrau as a function of the intensity over the entire dynamic range. At all intensities the noise is below the Poisson fluctuations shown as a black line. This means that the uncertainty of the data is limited by the Poisson fluctuations, *i.e.* the detector has the best possible data quality.

A very promising candidate for diffraction-limited light sources, due to its small pixel size, low noise, large dynamic range and high frame rate, is the Jungfrau detector. Fig. 3 ▶ shows schematically the pixel electronics of Jungfrau. The dynamic range covered by Jungfrau (similar for Gotthard and AGIPD) is 10^4^ 12 keV photons. The switching points are tuned such that at any point of the dynamic range the electronic noise is small compared with the Poisson fluctuations. Fig. 4 ▶ shows the measured noise as a function of the number of photons for Jungfrau. As can be seen, the noise is always negligible compared with the Poisson fluctuations. This means that the data are limited by photon statistics, *i.e.* the detector has the best possible data quality.

Jungfrau is currently being developed for SwissFEL and applications at synchrotrons and also in view of diffraction-limited light sources. It features a pixel size of 75 µm, and is a modular system with a module having about 500k pixels. Systems up to 16 Mpixel are foreseen for SwissFEL. A small noise of 120 electrons allows measuring of low energies down to about 2 keV. The maximal frame rate is 2 kHz which gives together with the dynamic range of 10^4^ photons a linear count rate capability of 20 MHz (for 12 keV photons) at a dead-time free mode of operation. By reduction of the acquisition time, *i.e.* introduction of a dead-time, a quasi infinite linear count rate capability can be achieved. Jungfrau is the first non-count-rate-limited large hybrid pixel detector. Applications like PX at synchrotrons today or ptychography at diffraction-limited light sources should therefore significantly profit from it.

For ptychography it is usually preferred to increase the scanning speed instead of increasing the statistics if the flux is high enough. This will most likely require an increase of the frame rate of Jungfrau to several kHz for optimal use for ptychography at diffraction-limited light sources and if combined with a multilayer monochromator to several 10 kHz.

### 2.3. Hybrid pixel detectors with smaller pixels   

**Figure 5:**
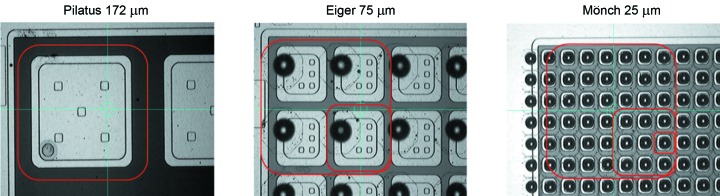
Zoom in on a silicon sensor with bumps for Pilatus (left), Eiger (middle) and Mönch (right). The photographs are to scale, *i.e.* the red squares indicate the pixel size of 172 µm from Pilatus (large), 75 µm from Eiger (medium) and 25 µm from Mönch (small). The 25 µm pixel size is close to the limit for the in-house bump-bonding process at the Paul Scherrer Institut.

The better focusing capabilities at diffraction-limited light sources might make smaller pixels desirable. Already today in PX for example peaks often fall in single pixels (Pilatus 172 µm pixel size). Since charge-integrating electronics do not require much space in the pixel layout, the pixel size can be reduced compared with single-photon-counting electronics. Also, charge-sharing effects where charge from one photon is split over several pixels is much less problematic for charge integrating detectors compared with single-photon-counting detectors. With a pixel size of 25 µm, Mönch (Dinapoli *et al.*, 2014 ▶; Cartier *et al.*, 2014 ▶) is currently the hybrid pixel detector with the smallest pixel size. Fig. 5 ▶ shows the pixel on the sensor for Pilatus, Eiger and Mönch. Similar to CCDs for soft X-rays it allows the determination of the absorption position of isolated photons with a resolution of 1–2 µm (Schubert *et al.*, 2012 ▶).

Mönch is currently a small prototype of 160 × 160 pixels for studying the feasibility of such small pixels for hybrid detectors. The main problems are the power consumption which needs to be dramatically reduced, the small area available for the electronics in a ASIC pixel and the narrow pitch for bump-bonding. The problems, however, all seem to be handable and a larger chip with 400 × 400 pixels is on the way and larger modules are foreseen. Mönch has a dynamic range of up to 1000 12 keV photons per pixel and integration time. It shows the way to smaller pixel sizes which might be required for the medium energy range due to the smaller beam sizes, for example for PX.

### 2.4. Detector for energies above 25 keV   

For energies above 25 keV, high-*Z* sensors like CdTe, CdZTe or GaAS offer a much higher efficiency than silicon sensors. CdTe- and CdZTe-based sensors, however, suffer from the so-called polarization effect which reduces their efficiency and usability at high photon rates (Hamann, 2013 ▶; Koenig *et al.*, 2013 ▶). Therefore, GaAs or thick silicon sensors (up to 1 mm) might be better for the expected high flux of diffraction-limited light sources. Also an indirect detection system using a scintillator and a CCD or charge-integrating system like Jungfrau might be an option.

## 3. Conclusions   

Diffraction-limited light sources will increase the coherent flux up to a few orders of magnitude, and reduce the horizontal beam divergence, which in turn will increase the brilliance. This will pose severe problems for detectors used in applications making use of coherence (CDI, ptychography) or those which benefit from the higher brilliance (XPCS). Single-photon-counting detectors in use at today’s third-generation light sources are often already count-rate-limited, and do not seem to be capable of coping with the high photon rates at a diffraction-limited light source.

Solutions for soft X-ray RIXS seem to be in hand. The very high count rates at brighter sources pose challenges for CCDs and more work is required for soft X-ray detectors.

For the medium energy range, X-ray hybrid charge-integrating pixel detectors with dynamic gain switching with increased frame rates (10 kHz or above) seem to be a good solution for the upcoming requirements.

The high frame rates and small pixel sizes will also increase the generated data volume by several orders of magnitude. The problems connected to this are not discussed here.
